# Prognostic value of HPV DNA status in cervical cancer before treatment: a systematic review and meta-analysis

**DOI:** 10.18632/oncotarget.18558

**Published:** 2017-06-16

**Authors:** Ping Li, Yue Tan, Li-Xia Zhu, Li-Na Zhou, Ping Zeng, Qin Liu, Min-Bin Chen, Ye Tian

**Affiliations:** ^1^ Department of Radiotherapy and Oncology, The Second Affiliated Hospital of Soochow University, Institute of Radiotherapy and Oncology, Soochow University, Suzhou 215004, Jiangsu Province, China; ^2^ Department of Radiotherapy and Oncology, Kunshan First People’s Hospital Affiliated to Jiangsu University, Kunshan 215300, Jiangsu Province, China; ^3^ Department of Gynecology, Kunshan First People’s Hospital Affiliated to Jiangsu University, Kunshan 215300, Jiangsu Province, China

**Keywords:** HPV, cervical cancer, prognosis, overall survival, disease free survival

## Abstract

**Background:**

Human papillomavirus (HPV), has been recognized as an vital preliminary event in the oncogenesis of cervical cancer. But the prognostic value is not well defined, because of past studies showing conflicting results. So we conducted this meta-analysis to evaluate whether HPV DNA status was associated with prognosis in cervical cancer.

**Materials and Methods:**

A total of 17 previously published eligible studies including 2,838 cases were identified and included in this meta-analysis. Positive HPV DNA was associated with good prognosis in patients with cervical cancer (overall survival [OS]: pooled hazard ratio (HR) = 0.610, 95% confidence interval (CI) = 0.457−0.814, *P* = 0.001; disease free survival [DFS]: pooled HR = 0.362, 95% CI = 0.252−0.519, *P* < 0.001). Furthermore, in subgroup analysis, the results revealed that the association between HPV DNA positive cervical cancers and better OS (pooled HR = 0.534, 95 % CI = 0.355–0.804, *P* = 0.003) in Mongoloid patients. Similarly, it existed in good OS (pooled HR = 0.628, 95 % CI 0.429−0.922, *P* = 0.017) and DFS (pooled HR = 0.355, 95% CI = 0.226−0.559, *P* < 0.001) in Caucasian patients.

**Conclusions:**

HPV DNA status in cervical cancer may be a useful prognostic biomarker before carcinomas are treated. However, larger sample sizes and more comprehensive studies are required in the future studies to verify our findings.

## INTRODUCTION

Cervical cancer is one of the most common malignancies in women worldwide. Based on the results of epidemiologic studies supported by basic experimental findings, the infection of human papillomavirus (HPV), especially high-risk HPV (HPVs 16, 18, 45, 56), has been recognized as an vital preliminary event in the oncogenesis of cervical cancer [1–3]. It has been reported that 95–100% of patients with invasive carcinoma of the cervix were infected with HPV [4].

Over the past decades, a large number of studies tried to evaluate the prognostic value of HPV status in cervical cancer, however, the exact mechanism about the prognostic factor of HPV was not totally understood. In HPV-positive cervical cancer, HPV sequences may be integrated in cells and thus enhance the expression of E6/E7 viral oncogenes to active oncogenes and alter cell growth [5]. While in HPV-negative cancers, some other oncogenes may take over E6-like and E7-like functions in tumorigenesis [6]. HPV-negative cervical cancer might represent a kind of biologically distinct tumors, which exists more frequently in node-positive cervical cancer [6] and is more often observed in such as adenocarcinomas, advanced stages [7]. Even so, more studies showed that no statistically significant difference was found between HPV-negative and HPV-positive cases in terms of clinical stage [4, 6–21]. In spite of some authors advocated the hypothesis that adenosquamous carcinoma was associated with a poor prognosis [22], it remained controversial [23]. For example, it was reported that HPV-negative cervical cancer had a higher incidence in squamous cell carcinoma (SCC), but not adenocarcinoma (AC) [15]. From then on, more and more studies have reported the prognostic value of HPV status in cervical cancer. Most studies have suggested HPV-negative cervical cancer carried a poorer prognosis than HPV-positive [4, 6–8, 12, 16, 17, 20]. However, some studies showed HPV DNA did not have any prognostic implication [9–11, 13, 18, 19, 21].

The prognostic significance of HPV status in cervical cancer before treatment have been got conflicted results and remained unclear. The size of the sample in each study was limited. Therefore, we performed this comprehensive metaanalysis to evaluate the prognostic value of HPV status in cervical cancer, so as to illustrate which kinds of patients may require careful follow-up for recurrence and need include additional treatment.

## RESULTS

### Demographic characteristics

**Figure 1:**
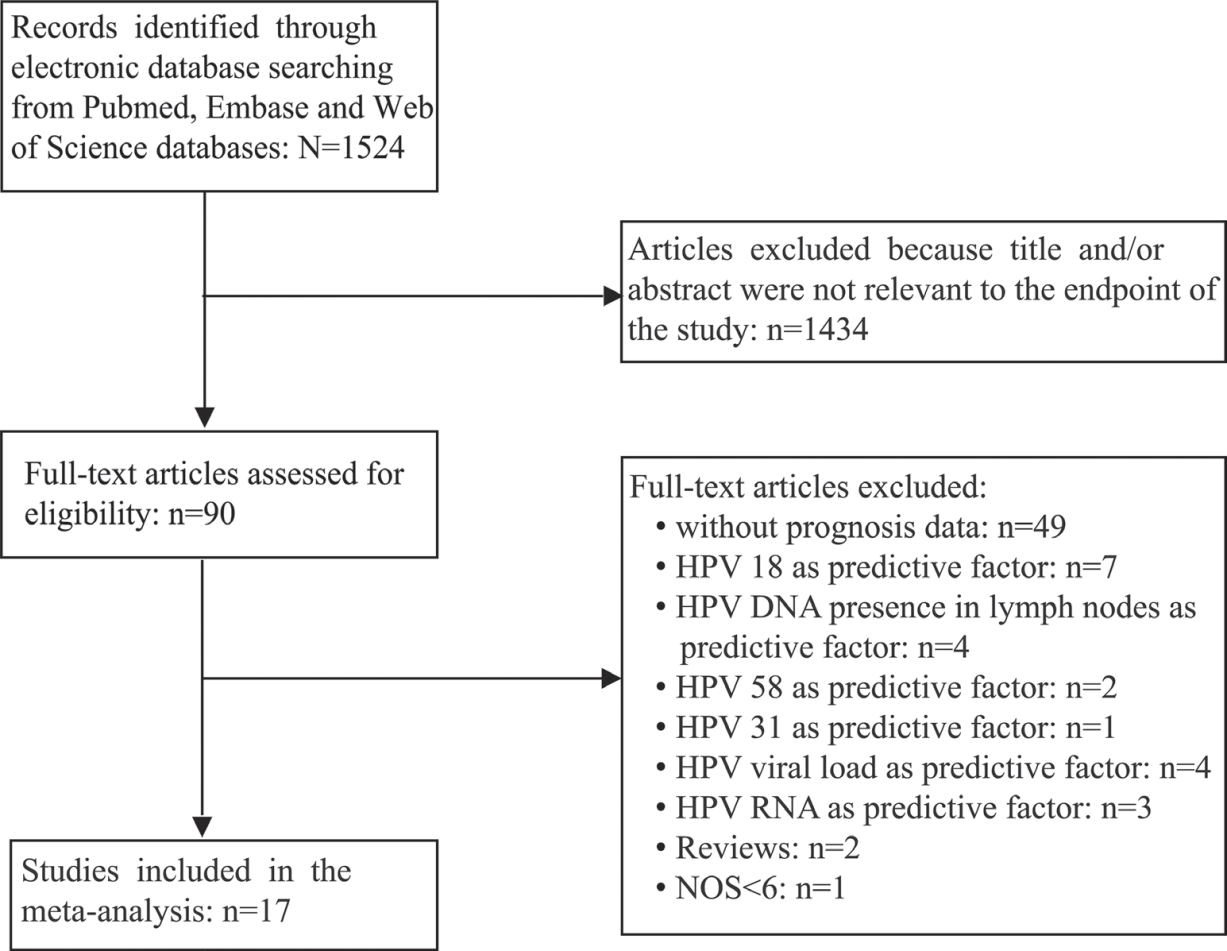
The flow chart of the selection process in our meta-analysis

A total of 1,524 articles were retrieved by a literature search of the PubMed, Embase, and Web of Science databases, using different combinations of key terms. As shown in the search flow diagram (Figure 1), 1,524 records were initially retrieved using the predefined search strategy. After browsing the retrieved titles and abstracts, 1,434 of them were excluded due to non-relevant endpoint provided. The remaining 90 records were downloaded as full-text and checked one by one carefully. Furthermore, another 70 studies were excluded, including 49 that had no prognosis data, 7 were prognosis about HPV 18, 4 were about predictive value of HPV DNA in cervical cancer lymph nodes, 2 were about clinical outcome of HPV 58 type, 1 was about HPV 31 prognosis data, 4 were involving prognosis of HPV viral load, 3 were about prognosis of HPV RNA, 2 were about reviews, 1 was excluded due to the quality score was less than 6. As a result, 17 published studies met the criteria for analysis between HPV DNA status and cervical cancer prognosis.

To assess the relevance between HPV DNA and cervical cancer prognosis, 2,838 patients met the inclusion standard were finally selected for the meta-analysis. The sample-size was with a wide range from 32 to 515. Among the 17 cohorts for HPV DNA status, 11 focused on OS, 3 focused on DFS and rest 3 focused on both OS and DFS; and after categorized by races, Caucasian (*n* = 11) became the major race of literatures including Europe (*n* = 10) and North America (*n* = 1), followed by Mongoloid (*n* = 6) including Japan (*n* = 3), China (*n* = 2) and Korea (*n* = 1), respectively.

### Evidence synthesis

**Figure 2:**
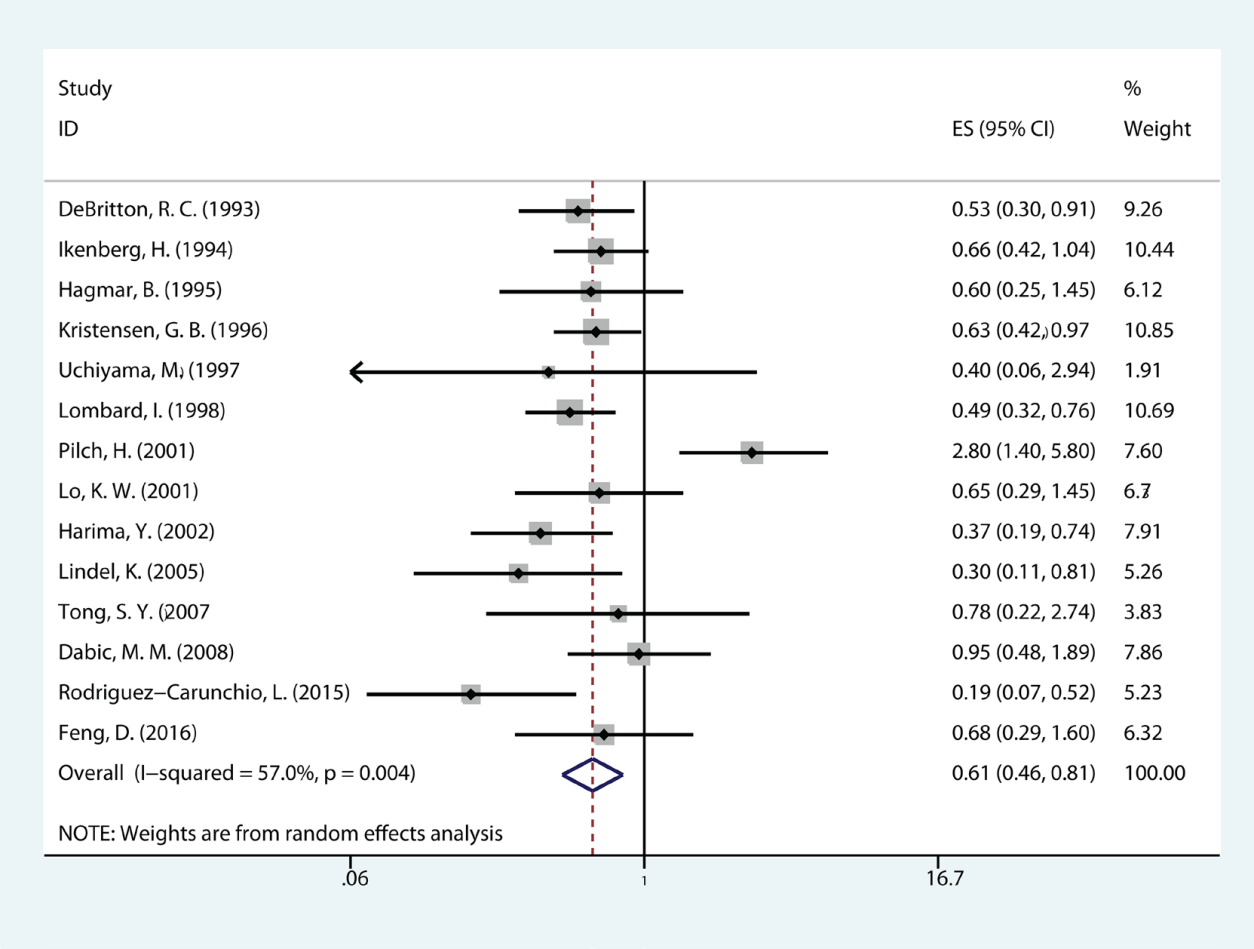
The correlation between HPV-positive and overall survival (OS) CCs

**Figure 3:**
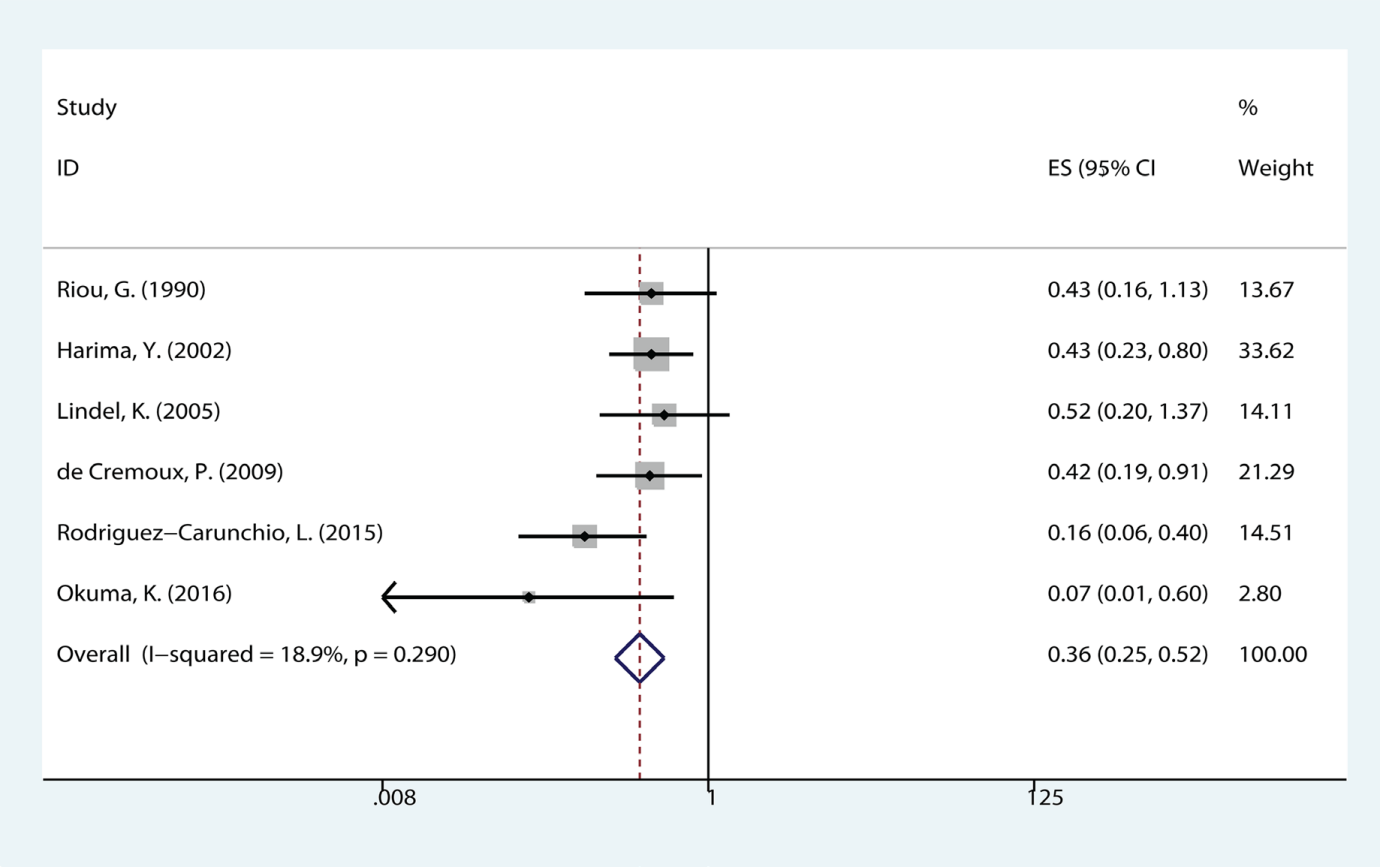
The correlation between HPV-positive and disease free survival (DFS) in CCs

The meta-analysis of HPV DNA status was based on two outcome endpoints: OS and DFS. For OS, 14 studies in total were included in the meta-analysis, inside which, given the fact that the *P* value of 0.004 and I^2^ values of 57% calculated from the heterogeneity test, a random-effects model was used. The results shown a significant association between HPV-positive cervical cancer and OS (pooled HR = 0.610, 95% CI = 0.457–0.814, *P* = 0.001) (Figure 2). There were 6 studies included in the meta-analysis of DFS, of which a fixed effects model was utilized to calculate the pooled hazard ratio (HR) and 95% confidence interval (CI) on account of the *P* value of 0.290 and *I*^2^ values of 18.9% reported by the heterogeneity test. The results suggested that HPV-positive cervical cancer had significantly better DFS (pooled HR = 0.362, 95% CI = 0.252–0.519, *P* < 0.000) (Figure 3). Subgroup study was then performed, and the results suggested that HPV-positive cervical cancer was associated with good OS in Mongoloid patients (pooled HR =0.534, 95 % CI 0.355–0.804, *P* = 0.003), as well as in Caucasian (OS: pooled HR = 0.628, 95 % CI 0.429–0.922, *P* = 0.017; DFS: pooled HR = 0.355, 95% CI = 0.226–0.559, *P* < 0.000).

### Publication bias and sensitivity analysis

**Figure 4:**
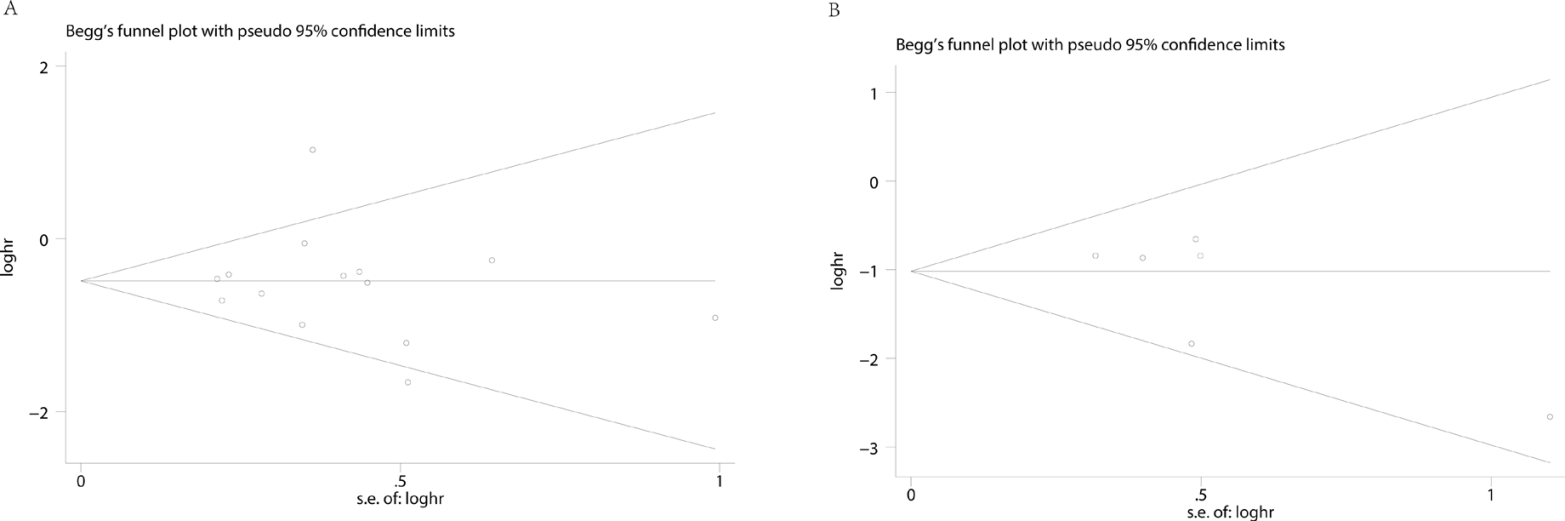
Begg’s funnel plots for the studies involved in the meta-analysis **(A)** overall survival (**B**) disease free survival.

**Figure 5:**
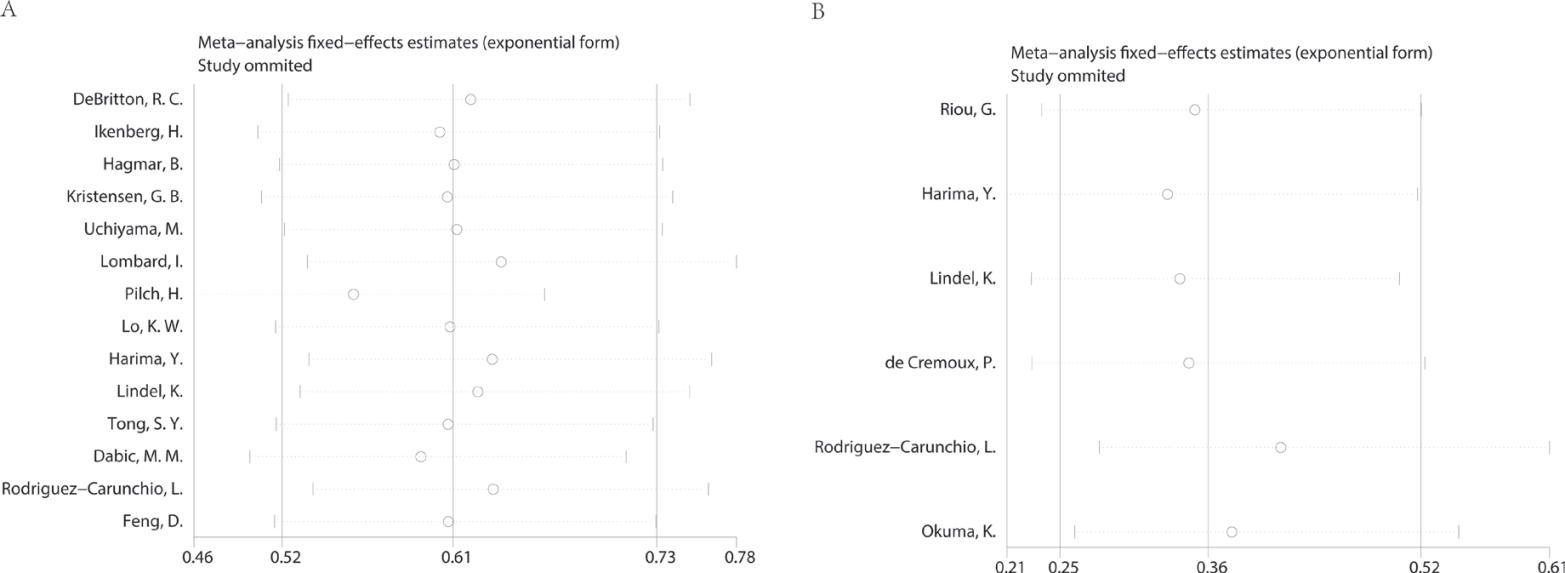
Sensitivity analysis of the meta-analysis **(A)** overall survival (**B**) disease free survival.

Begg’s funnel plot and Egger’s test were applied to assess the publication in this meta-analysis. The shapes of the funnel plots showed no evidence of obvious heterogeneity. Egger’s tests, following OS, DFS in cervical cancer about HPV DNA (*P* = 0.792; *P* = 0.171) (Figure 4), revealed no publication bias. Sensitivity analyses were further utilized to determine the influence of the results described above. No individual study dominated this meta-analysis. Removing any single study had no significant effect on the final conclusion (Figure 5).

## DISCUSSION

HPV has been identified as the key causative agent for the development of cervical cancer. Most studies had determined whether HPV DNA status before treatment was a prognostic factor in cervical carcinoma patients. However, as large centers were hardly able to recruit patients with large sample size, the conclusions from every center remained non-comprehensive.

To our knowledge, current meta-analysis is the first complete overview showing the prognosis significant of HPV DNA status in cervical cancer. We evaluated the survival data of 2,838 cervical cancer patients about HPV DNA status and included in total 17 different studies systematically. As a whole, the results clearly determine that positive HPV DNA is a better prognostic factor in cervical cancer, with better DFS (pooled HR = 0.362, 95% CI = 0.252–0.519, *P* < 0.000) and better OS (pooled HR =0.610, 95% CI = 0.457–0.814, *P* = 0.001). In subgroup analysis, the results revealed that the association between HPV DNA positive cervical cancer patients and better OS (pooled HR = 0.534, 95% CI 0.355–0.804, *P* = 0.003) in Mongoloid patients. Similarly, it existed in good OS (pooled HR = 0.628, 95% CI 0.429–0.922, *P* = 0.017) and DFS (pooled HR = 0.355, 95% CI = 0.226–0.559, *P* < 0.000) in Caucasian patients.

Actually, as we all known, FIGO stage, lymph node status, primary tumor size, stromal invasion depth, lymph-vascular space invasion and the vaginal margins remain the common prognostic parameters for cervical cancer [24]. In current meta-analysis, it reveals that positive HPV DNA may have good OS and DFS in patients with cervical cancer. Similar meta-analysis results have also been reported in HPV-positive head and neck squamous cell carcinoma [25, 26]. It is pretty clear that due to its significant prognostic effects, HPV status of cervical cancer now should be considered as a prognostic marker before treatment. HPV negative primary cancers, which showed a great potential to metastasize, was found existing more aggressive p53 mutations than HPV positive in the normal development process, resulting in a more severe deregulation of normal growth control and a worse prognosis [6, 27]. P53 mutations detected may suggest the poor prognosis in HPV negative cervical cancer in one way. Despite our attempts to perform the explanation, the reason of different prognosis in cervical cancer is not totally clear recently. More research from the perspective of molecular biology should be carried out to explore the complicated carcinogenesis mechanism in HPV-positive cervical cancer.

As illustrating our results, some limitations were existed in current meta-analysis. First, we limited the search of studies performing in English, and did not search conference proceedings or books, which may introduce publication bias to meta-analysis. We tried to recruit all relevant data and additional unpublished information, but some missing data were unavoidable. Second, most included studies were reported as retrospective studies, which were more likely to be published if they had positive results than negative results. Third, the heterogeneity may increase due to vary HPV genotypes existence in different center, but high-risk HPV types are dominated in cervical cancer patients among individual studies. Even though this meta-analysis has these restrictions, it still has several strengths. Initially, all the limited subsets have provided 2,838 patients, which represented a substantial number of cases and increased the statistical power in the analysis significantly. Besides, no publication biases were detected, which indicated the pooled results may be impartial.

This study is the first meta-analysis to assess the prognostic significance of HPV status in cervical cancer before treatment. Our data support the positive HPV DNA is a better prognostic factor in cervical cancer. Patients with negative HPV DNA may require careful follow-up for recurrence and need include additional treatment. However, larger sample sizes and more comprehensive study designs are required in the future studies to verify our finding.

## MATERIALS AND METHODS

### Publication search

A comprehensive literature search of the electronic databases PubMed, EMBASE and Web of Science databases was performed up to November 28, 2016, with the search terms: ‘cervical cancer’, ‘cervical carcinoma’, ‘carcinoma of cervix’, ‘HPV’ and ‘prognosis’. All potentially eligible studies were retrieved and their bibliographies were carefully scanned to identify other eligible studies. Extra studies were identified by a hand search of the references cited in the original studies. When multiple studies of the same patient population were identified, we included the published report with the largest sample size. Only studies published in English were included in this meta-analysis.

### Inclusion and exclusion criteria

Studies included in this meta-analysis had to meet all of the following criteria: (a) evaluation of HPV DNA status for predicting prognosis in cervical cancer, (b) provide hazard ratios (HRs) with 95% confidence intervals (CIs) or enable calculation of these statistics from the data presented, (c) classify HPV status as ‘positive’ and ‘negative’.

Exclusion criteria were: (a) literatures were published as letters, editorials, abstracts, reviews, case reports and expert opinions; (b) experiments were performed *in vitro* or *in vivo*, but not based on patients; (c) articles were without the HRs, 95% CI, or not dealing with OS, DFS, or the K-M survival curves; (d) the follow-up duration was shorter than 3 years.

### Data extraction

Information was carefully and independently extracted from all eligible publications by two authors using a standardized form. Disagreement was resolved through independently extracting data from the original article by the third author, and consensus was reached by discussions. The meta-analysis of this study was based on two outcome endpoints: OS and DFS. As stated by the inclusion and exclusion criteria above, the following items were extracted from each study, including the first author’s name, publication year, country of origin, number of patients analyzed, tumor stage, clinicopathologic factors, source of tissue, HPV detection method, HPV genotype detected, OS and DFS. The main features of these eligible studies were summarized in Table 1. For the articles in which prognosis was plotted only as the Kaplan-Meier curves, the Engauge Digitizer V4.1 was used to extract survival data, then applied Tierney’s method to estimate the HRs and 95% CIs [28]. All studies were assessed by Newcastle-Ottawa Scale (NOS) [29]. The quality scores ranging from 6 to 9, suggested that the methodological quality was high.

**Table 1:** Characteristics of studies included in the meta-analysis

author	year	country	No of cases	FIGO stage	histology	source	method of detecting HPV	HPV genotype detected	endpoints	NOS
Riou G. [6]	1990	France	106	Ib–IIb	SCC, AC	fresh	SBH + PCR	16, 18, 31, 33, 35, 39, 6, 11 and 42	DFS	7
DeBritton RC. [8]	1993	Panama(muti-center)	178	I–IV	SCC, AC, ASC	fresh	SBH + PCR	16, 18 and 33	OS	8
Ikenberg H. [9]	1994	Germany	205	Ib–IV	SCC, AC, ASC	fresh	SBH + PCR	16, 18, 31, 35, 33 and type unknown	OS	8
Hagmar B. [10]	1995	Sweden	97	I–IV	SCC	paraffin	PCR	16, 18, 31, 33, and type unknown	OS	8
Kristensen GB. [11]	1996	Norway	223	I–IV	SCC, AC, ASC	fresh	PCR	16, 18, 33 and type unknown	OS	6
Uchiyama M. [12]	1997	Japan	32	0–IV	AC, ASC	formalin	PCR	16, 18 and other types	OS	6
Lombard I. [13]	1998	France	297	I–IV	SCC, AC, ASC	fresh	SBH + PCR	16, 18, 31, 33, 58, 35, 45 and 52	OS	7
Pilch H. [14]	2001	Germany	203	I–II	SCC, AC, ASC	paraffin	PCR	16, 18 and rare HPV types	OS	8
Keith WK. Lo [15]	2001	China	121	I–IV	SCC, AC	fresh	PCR	16, 18, 31, 33, 52, 53, 56, 58 and type unknown	OS	9
Harima Y. [16]	2002	Japan	84	Ib–IVb	SCC, AC	fresh	PCR	16, 18, 6, 31, 33, 52 and 58	OS, DFS	7
Lindel K. [17]	2005	Switzerland	40	I–IV	SCC, AC, ASC	paraffin	PCR	16, 6, 31 and 33	OS, DFS	7
Tong SY. [18]	2007	Korea	97	I–IV	SCC, AC, ASC	fresh	HDC + PCR	16, 18, 35, 33, 58, 66, 68, 31, 52 and 56	OS	8
Dabic MM. [19]	2008	Croatia	51	I–IV	AC	paraffin	PCR	16, 18, 51, 31, 33 and 45	OS	7
de Cremoux P. [20]	2009	France	515	I–IV	SCC, AC	fresh	PCR	16, 18, 45, 31, 33, 35, 39, 52, 53, 58, 59 and 73	DFS	6
Rodriguez-Carunchio L. [7]	2015	Spain	136	I–IV	SCC, AC, ASC	paraffin	PCR, HC2	16, 18, 45,and 68	OS, DFS	7
Okuma K. [4]	2016	Japan	71	I–IV	SCC, AC, ASC	fresh	PCR	16, 18, 31, 33,39, 44, 52, 56, 58, 59, 66 and 68	DFS	8
Feng D. [21]	2016	China	122	I–III	SCC, AC, ASC	paraffin	PCR	16, 18 and other types	OS	7

#SCC: Squamous cell carcinoma; AC: Adenocarcinoma; ASC: adenosquamouscarcinoma.

*SBH: Southern blot hybridization; PCR: polymerase chain reaction; HC2: Hybrid capture II assay; HDC: HPV DNA chip.

### Statistical analysis

The data collected from each qualified paper was used to evaluate the association between HPV DNA status and cervical cancer prognosis through meta-analysis. Pooled HRs and 95% CIs for their outcome endpoints (OS and DFS) were calculated. Subgroup analysis was performed when there were at least three studies in each subgroup. Statistical heterogeneity was assessed using Cochran’s Q test and Higgins’s I^2^ statistic [30], *P* value > 0.10 and *I*^2^ < 40% suggested a lack of heterogeneity among studies. According to the absence or presence of heterogeneity, random effects model or fixed effects model was used to merge HR, respectively.

Funnel plots and the Egger’s test were employed to evaluate the possible publication bias [31]. If a publication bias did exist, its influence on the overall effect was assessed by the Duval and Tweedie’s trim and fill method [32]. Sensitivity analysis was also performed to estimate if certain single article could influence the overall result. Statistical analyses were conducted using Stata 14.0 (StataCorp, College Station, TX). *P* values for comparisons were two-tailed and statistical signiﬁcance was deﬁned as *p* < 0.05 for all tests, except those ones with heterogeneity.
